# Cinnamaldehyde Exerts Its Antifungal Activity by Disrupting the Cell Wall Integrity of *Geotrichum citri-aurantii*

**DOI:** 10.3389/fmicb.2019.00055

**Published:** 2019-01-30

**Authors:** Qiuli OuYang, Xiaofang Duan, Lu Li, Nengguo Tao

**Affiliations:** School of Chemical Engineering, Xiangtan University, Xiangtan, China

**Keywords:** *Geotrichum citri-aurantii*, Cinnamaldehyde, cell membrane, cell wall, antifungal mechanism

## Abstract

Our previous study showed that cinnamaldehyde (CA) significantly inhibited the mycelial growth of *Geotrichum citri-aurantii*, one of the main postharvest pathogens in citrus fruits. This study investigated the antifungal mechanism of CA against *G. citri-aurantii*. Scanning electron microscopy (SEM) and transmission electron microscopy (TEM) images showed that CA treatment led to clear morphological changes in the cell walls and membranes of *G. citri-aurantii*. However, the membrane integrity, total lipids and ergosterol contents were not apparently affected by CA treatment. Notably, the extracellular alkaline phosphatase (AKP) activity was increased after CA treatment, suggesting impairment in cell wall permeability. A weakened fluorescence in the cell wall, a decrease in the chitin contents, and changes of ten genes involved in cell wall integrity were also observed. These results suggested that CA may exhibit its antifungal activity against *G. citri-aurantii* by interfering the build of cell wall and therefore lead to the damage of cell wall permeability and integrity.

## Introduction

Worldwide citrus sour rot caused by *Geotrichum citri-aurantii*, which is less common than green and blue molds, has been reported as an important postharvest disease of citrus fruits, particularly during periods of high rainfall (McKay et al., 2012; Zhou et al., 2014). Currently, sour rot cannot be actively controlled by the registered fungicides imazalil, thiabendazole, pyrimethanil and fludioxonil and can only be partially controlled with the commercial fungicides sodium *o*-phenylphenate (SOPP) and propiconazole (McKay et al., 2012). However, the concentration of SOPP required to effectively manage sour rot can result in the development of oleocellosis and darkening of the fruit rind (Regnier et al., 2014; Zhou et al., 2014). Therefore, the use of essential oils represents a promising strategy for the management of citrus sour rot due to their effective and commercial characteristics.

Many studies highlight the feasibility of applying essential oils for postharvest disease control in citrus due to their notable antifungal activity against *G. citri-aurantii* and substantially lower risk for the development of resistance (Talibi et al., 2012; Regnier et al., 2014; Wuryatmo et al., 2014). Cinnamaldehyde (CA), a major constituent of cinnamon essential oils, was demonstrated to have potent antifungal activities against a wide variety of fungi (Bang et al., 2000; Wang et al., 2005; Wu et al., 2017; Duan et al., 2018). Our previous study showed that CA significantly inhibited the mycelial growth of *G. citri-aurantii* with a minimum inhibitory concentration (MIC) and minimum fungicidal concentration (MFC) of 0.50 mL/L (Wu et al., 2017). Furthermore, wax + CA treatments had strong inhibition effects on sour rot and green mold decay during storage time and induced significant citrus fruit defense responses against the pathogens (Wu et al., 2017; Duan et al., 2018). Therefore, CA may be a potential candidate as an effective and environmentally benign citrus preservative.

Until now, the antifungal mechanism of CA was widely investigated but there were no conclusive findings. Previous studies reported that CA acts by perturbing the cell membrane, acting as an ATPase inhibitor, inhibiting enzymes involved in cytokine interaction, or inhibiting cell wall biosynthesis (Smid et al., 1996; Bang et al., 2000; Xie et al., 2004; Shreaz et al., 2011, 2013; Xing et al., 2014). In a study on *Saccharomyces cerevisiae, trans*-cinnamaldehyde caused a partial collapse of the integrity of the cytoplasmic membrane, leading to the excessive leakage of metabolites and enzymes of the cell and finally loss of viability (Smid et al., 1996). However, Bang et al. (2000) found that CA inhibited the growth of *S. cerevisiae* cells because of its ability to inhibit cell wall-synthesizing enzymes, *β*-(1,3)-glucan synthase and chitin synthase I. In another study, CA inhibited the growth of *Phytophthora capsici* by disturbing calcium homeostasis (Hu et al., 2013). Xie et al. (2004) and Xing et al. (2014) demonstrated that CA significantly inhibited the growth of *Aspergillus flavus* and *Fusarium verticillioides* by causing irreversible deleterious morphological and ultrastructural alterations, such as the lack of cytoplasmic contents, loss of integrity and rigidity of the cell wall, disruption of the plasma membrane, and destruction of mitochondria. Recently, Shreaz et al. (2011) demonstrated that CA exerted its antifungal activity by targeting the sterol biosynthesis of *Candida*, whereas in another study, the immediate effect of CA against *Candida* may originate from the inhibition of PM-ATPase and a decrease of pHi, or may contribute to the depletion of NADPH (Shreaz et al., 2013). Nevertheless, to our knowledge, there are no reports on the underlying mechanism of the antifungal action of CA against *G. citri-aurantii*. Thus, the inhibitory mechanism of CA against *G. citri-aurantii* requires further studies.

The objective of this research was to reveal the antifungal mechanism of CA against *G. citri-aurantii* by determining the following: (i) the morphology and ultrastructure of cell membranes using SEM and TEM, (ii) the plasma membrane integrity and the total lipid and ergosterol contents, (iii) the extracellular alkaline phosphatase (AKP) activity, the calcofluor white stain results and chitin contents, (iv) and the expression levels of genes involved in cell walls.

## Materials and Methods

### Fungal Strain

*Geotrichum citri-aurantii* was provided by the Department of Biotechnology and Food Engineering, Xiangtan University, Xiangtan, China. The fungal pathogen *G. citri-aurantii* used in this study was isolated from infected citrus fruit (Zhou et al., 2014) and cultured on potato dextrose agar (PDA) at 28 ± 2°C. A spore suspension (5 × 10^5^ spores/mL) in potato dextrose broth (PDB) was prepared using a hemocytometer.

### Preparation of CA Treatments

For solid culture, CA (Sigma, St. Louis, MO, United States) was added into PDA (with 0.05% Tween-80) for a final concentration of 0.25 μL/mL (1/2MIC). A 6 mm inoculum disk cut with a cork borer from the leading edge of the fungal culture on PDA plates was placed at the center of each new plate. Culture plates were then incubated at 28 ± 2°C for 4 days.

For liquid culture, CA was added into PDB for a final concentration of 0.25 μL/mL (1/2MIC). Two hundred milliliters spore suspension (5 × 10^5^ spores/mL) were added into 40 mL PDB containing CA and incubated in a moist chamber with 160 r/min at 28 ± 2 °C for 0, 30, 60, and 120 min.

### Scanning Electron Microscopy (SEM)

The 4-day-old fungal culture described above was directly examined with a JEOL JSM-6360LV SEM (JEOL, Tokyo, Japan). The hyphae grown on PDA without CA were used as a control. The procedures for the SEM observation were described in our previous study (Zhou et al., 2014).

### Transmission Electron Microscopy (TEM)

The 4-day-old fungal culture described above was directly examined with a transmission electron microscope (JEM-1230; JEOL Ltd., Tokyo, Japan) operated at an accelerating voltage of 80 kV. The hyphae grown on PDA without CA were used as a control. The procedures for the TEM observation were described in our previous study (Tao et al., 2014).

### Assay for Plasma Membrane Integrity

Plasma membrane integrity of the *G. citri-aurantii* with different CA treatments in PDB described above was analyzed by propidium iodide (PI) staining coupled with an F97 PRO fluorescence spectrophotometer (Lengguang Technology, Shanghai, China) (OuYang et al., 2018).

### Determination of Ergosterol Content

Total ergosterol contents of *G. citri-aurantii* cells with different CA treatments in PDB described above were determined using the HPLC method (OuYang et al., 2016). The fungal culture on PDA without CA was used as a control. The samples were dried with a vacuum freeze drier for 4 h. About 0.1 g of dry mycelia were homogenized with liquid nitrogen and saponified by adding 4 mL of freshly prepared 30% (w/v) methanolic KOH and 8 mL of absolute ethanol at 90 °C for 2 h. The mixtures were extracted with 3 mL petroleum ether for three times and washed by saturated NaCl solution twice. The samples were then vacuum concentrated and dissolved in 10 mL absolute ethanol.

HPLC was conducted on a Shimadzu LC-20AT liquid chromatography system (Shimadzu Scientific Instrument, Japan) equipped with a model LC-20AT solvent delivery system, a model SPD-M20A photo diode array detection system, and an Empower Chromatography Manager. The sample extracts were separated and analyzed by using a C18 column (250 mm × 4.6 mm, 5 mm) at room temperature. The mobile phase consisted of solvent A (methanol). The flow rate was 1.0 mL/min. Chromatographic peaks were identified by comparing the retention times and spectra against the known standard. The detecting wavelength was set at 282 nm. Aliquots of 20 mL were directly injected into the HPLC for the determination. All injections were repeated three times.

### Determination of Total Lipid Content

Total lipid contents of *G. citri-aurantii* cells with different CA treatments in PDB described above were determined using the phosphovanillin method (Tao et al., 2014). The fungal culture in PDB without CA was used as a control. The samples were dried with a vacuum freeze drier for 4 h. About 0.1 g of dry mycelia were homogenized with liquid nitrogen and extracted with 4.0 mL of methanol/chloroform/water mixture (2:1:0.8, v/v/v) in a clean dry test tube with vigorous shaking for 30 min. The tubes were centrifuged at 4000 g for 10 min. The lower phase containing lipids was thoroughly mixed with 0.2 mL saline solution and centrifuged at 4000 g for 10 min. Then, an aliquot of 0.2 mL chloroform and lipid mixture was transferred to a novel tube and 0.5 mL H_2_SO_4_ was added, heated for 10 min in a boiling water bath. After that, 3 mL phosphovanillin was added and shake vigorously, and then incubated at room temperature for 10 min. The absorbance at 520 nm was utilized to calculate total lipid contents from the standard calibration curve using cholesterol as a standard.

### Assays for Extracellular Alkaline Phosphatase (AKP) Activity

The extracellular AKP activities of *G. citri-aurantii* mycelia with different CA treatments in PDB described above were assayed by a UV-2450 UV/Vis spectrophotometer [Shimadzu (China) Co., Ltd., Shanghai, China] using the AKP kit (Solarbio Science and Technology Co., Ltd., Beijing, China) following the instructions. The fungal culture in PDB without CA was used as a control. Each experiment was repeated three times. The enzyme activity was expressed as U/g prot.

### The Effect of CA on the Cell Wall Integrity of *G. citri-aurantii*

The effects of CA on the cell wall integrity of *G. citri-aurantii* were analyzed by calcofluor white (Sigma, St. Louis, MO, United States) staining coupled with fluorescence microscopy. The 2-day-old mycelia were collected from 50 mL PDB containing 1/2MIC CA and centrifuged at 4000 g for 10 min. The collected mycelia were stained with 10 μL of calcofluor white stain after the addition of 10 μL KOH (10%) following the manufacturer’s instructions. Samples were observed with a fluorescence microscope (Nikon ECLIPSE TS100, Japan). The fungal culture in PDB without CA was used as a control.

### Determination of the Chitin Contents

The chitin contents of *G. citri-aurantii* cells with different CA treatments in PDB described above were determined by the following steps. The samples were dried and ground into a powder. Then, the powders (W_1_) were treated with a saturated KOH solution at 160°C for at least 15 min until the products became a transparent thin film. Then, the products were poured onto filter paper and rinsed slowly with distilled water. The extracts were successively dehydrated by 95 and 100% alcohol, weighing again as W_2_. The chitin contents were calculated by the following formula (Jiang, 2015):

$$\mathrm{chitin}\;\mathrm{content}\;(\%)=W_{2}/W_{1}\times 1.26\times 100\%$$

where 1.26 is the conversion factor.

### Real-Time Fluorescence Quantitative PCR (RTFQ-PCR) Analysis

The *G. citri-aurantii* cells treated for 0, 30, and 60 min were collected from PDB containing 1/2MIC of CA. RNA was extracted from *G. citri-aurantii* cells using the Trizol reagent (Invitrogen, United States) following the manufacturer’s instructions. Two micrograms of DNAfree RNA were used for reverse transcription by M-MLV (Promega, United States) with oligo dT18. RTFQ-PCR was performed on a BIO-RAD CFX Connect Thermal Cycler (Bio-Rad, CA, United States) using the FastStart Universal SYBR Green Master (Roche, Switzerland). The primers for the tested genes were designed base on our RNA-Seq data of *G. citri-aurantii* (data not shown) and listed in Table 1. RTFQ-PCR was programmed as follows: 95°C for 10 min followed by 40 cycles of 95°C for 15 s and 60°C for 1 min. The 2^-ΔΔCT^ method was used to quantify the value of each sample using the *r-actin* gene as an internal reference (Livak and Schmittgen, 2001).

**Table 1:** Primer pair sequences designed for validation of differentially expressed genes in control and 1/2MIC CA treatment of *G. citri-aurantii* using Real-time Fluorescence Quantitative PCR (RTFQ-PCR).

Gene name	Primer sequence (5′-3′, forward/reverse)
*r-actin*	TTACGCCGGTTTCTCCCTCC
	GACGATTTCACGCTCGGCAG
*CHS5*	GCGCCAAACCGCTACAAGAC
	TGATCCATTCAGGGCGCACG
*CHS2*	TACCAGCTTCTCCGGGCTTG
	GCAACACACCACAGGACGAC
*chsA*	CCGCCTATTCCCGATACCGC
	CCGCAAGAACAGCCACAGAC
*chsB*	GTTCTCATGGCGCTTGGCTC
	GTGCCAGTTGCAGAAAGCGT
*chsG*	GCTGGGAAATCGGCCAAAGG
	CGGACCGCCAGGTAAAATGC
*CHI1*	CGCGTGCCAAAGCTGACTAC
	GCGCCGACAAGGGATTTCTG
*ChiA1*	CGTTGTTCCCAAGCCAACCC
	CACAGAAGCGCTGGCAGAGT
*UAP1*	ATGTGGACCTAACCACGGGC
	ACGATACACCTCCAGCGAGC
*Glu1*	TTATCCATGCGCTCCCCCAC
	CGCTGATAGAGTCACCGCCA
*Glu2*	TACTGGATGGTCAACGGCGG
	TGTGGTGCCAGAGTGGTCAG


### Statistical Analysis

All data were expressed as the mean ± standard deviation of three independent replicates. A one-way analysis of variance (ANOVA) followed by Duncan’s test was performed to test the significance of differences between means obtained among the treatments at the 5% level of significance using the SPSS statistical software package release 16.0 (SPSS Inc., Chicago, IL, United States).

## Results

### Morphology Change of CA Treated *G. citri-aurantii* by SEM

**FIGURE 1:**
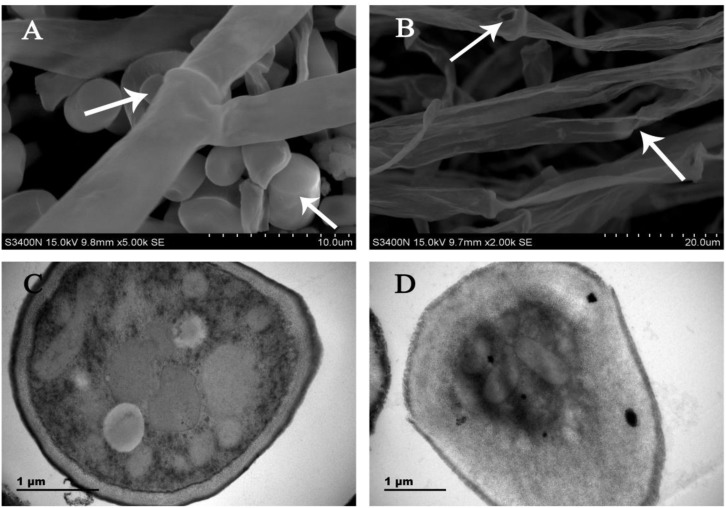
SEM images of **(A)** untreated control culture of *G. citri-aurantii* for 4 days; **(B)** culture after incubation with the CA for 4 days. TEM images of **(C)** untreated control culture of *G. citri-aurantii* for 4 days; **(D)** culture after incubation with the CA for 4 days.

Analysis of SEM images showed that the control samples exhibited normal morphology with regular, homogenous and robust hyphae of a constant diameter and a smooth surface (Figure 1A). However, the mycelia after CA treatment showed considerable alterations, which appeared as a damaged surface with rips and partly squashed, distorted, and shriveled mycelia (Figure 1B).

### Cell Wall Damage of CA Treated *G. citri-aurantii* by TEM

Analysis of TEM images showed that the untreated healthy mycelia showed a cell wall with uniform layers, defined plasma-membrane and periplasm region with normal thickness. The cytoplasm was clear, and well-organized mitochondria were scattered throughout the cytoplasm (Figure 1C). By contrast, CA damaged the cell wall and cell membrane of *G. citri-aurantii*. The hyphal wall was disrupted and even disappeared in some regions. The plasmalemma appeared faint, undefined and irregular. Discrete cytoplasmic alterations, including a very dense cytoplasm and a mass of disorganized structures, were also observed (Figure 1D).

### Effect of CA on the Plasma Membrane Integrity of *G. citri-aurantii*

**FIGURE 2:**
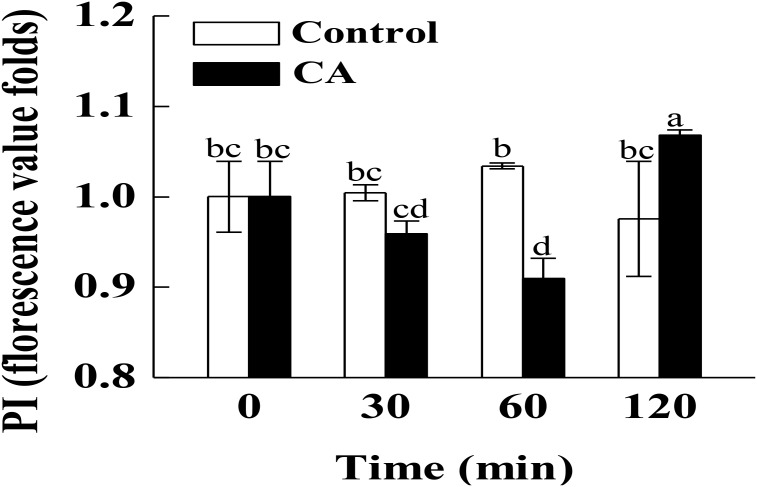
Effects of CA on the plasma membrane integrity of *G. citri-aurantii* mycelia. The data presented are the means of pooled data. Error bars indicate the SDs of the means (*n* = 3). Bars with different lower-case letters between different groups indicate significant differences according to duncan’s test (*P* < 0.05).

According to the PI staining results, the hyphae after 120 min of CA treatment presented a high fluorescence intensity (Figure 2, *P* < 0.05), which was generated by the damage to the plasma membrane integrity. However, the fluorescence intensity in CA treated samples before 60 min of exposure were lower than in the control, suggesting that the plasma membrane integrity was not damaged during this period.

### Effect of CA on the Total Lipid Content of *G. citri-aurantii*

**FIGURE 3:**
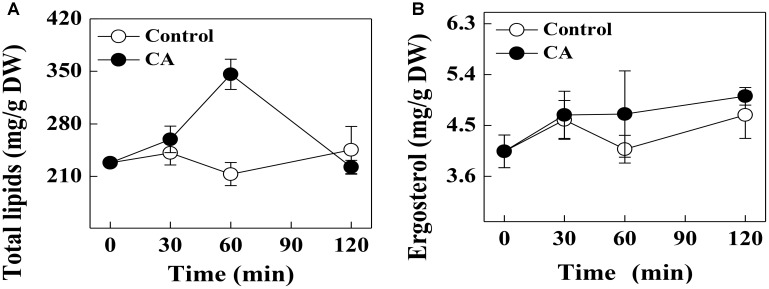
Effects of CA on **(A)** the total lipids contents and **(B)** ergosterol contents of *G. citri-aurantii* mycelia. The data presented are the means of pooled data. Error bars indicate the SDs of the means (*n* = 3).

The effect of CA on the total lipid content of *G. citri-aurantii* cells is shown in Figure 3A. The total lipid contents in control samples remained stable during the incubation time, whereas the CA treatment significantly induced the accumulation of total lipid contents at 60 min of exposure. The total lipid content of the CA treatment at 60 min was 346.0 ± 20.1 mg/g DW, which was significantly higher than the control (213.1 ± 15.5 mg/g DW) (*P* < 0.05). Nevertheless, the total lipid contents of CA treatment decreased after 60 min of exposure and no difference in the total lipid contents were observed between the 120 min treatment and the control.

### Effect of CA on the Ergosterol Content of *G. citri-aurantii*

There was no significant difference in the ergosterol contents of the CA treatments and control during the entire period. At 120 min of exposure, the ergosterol contents in the treated and control groups were 5.01 ± 0.15 and 4.69 ± 0.42 mg/g DW, respectively (Figure 3B).

### Effect of CA on the Extracellular AKP Activity of *G. citri-aurantii*

**FIGURE 4:**
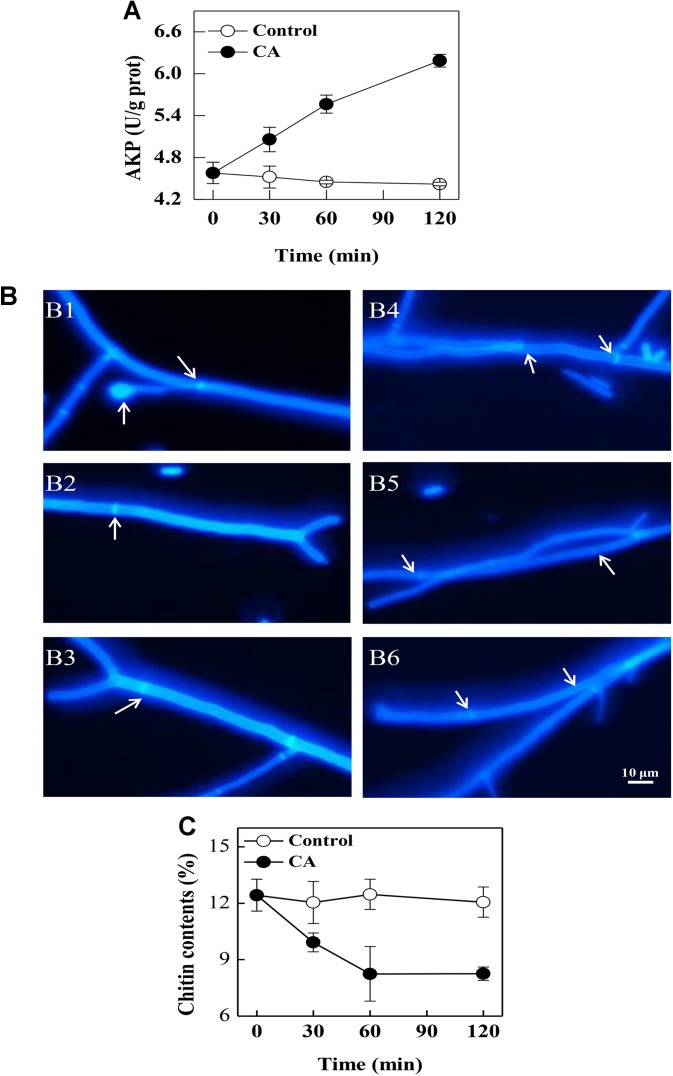
The effects of CA on the cell walls of *G. citri-aurantii*. **(A)** The extracellular AKP activity of *G. citri-aurantii*; **(B)** *G. citri-aurantii* treated with the 1/2MIC CA observed under a fluorescence microscopy after staining with calcofluor white stain (×100 magnification): **(B1–B3)** control group treated for 30, 60, and 120 min, respectively; **(B4–B6)** 1/2MIC CA group treated for 30, 60, and 120 min, respectively. **(C)** The chitin content of *G. citri-aurantii*. The data presented are the means of pooled data. Error bars indicate the SDs of the means (*n* = 3).

At 30 min of exposure, the extracellular AKP activity in the CA treatment was 5.06 ± 0.18 U/g prot, which was significantly higher than the control (4.52 ± 0.16 U/g prot). This change became more evident with increasing exposure time (*P* < 0.05). At 120 min of exposure, the extracellular AKP activity in the CA treatment was 6.18 ± 0.09 U/g prot, which was significantly higher than the control (4.42 ± 0.03 U/g prot) (Figure 4A).

### Effect of CA on the Cell Wall of *G. citri-aurantii*

As shown in Figure 4B, the fluorescence of the control cells was uniformly distributed, and the septa were visible with brighter fluorescent lines because septa are particularly rich in chitin (Figure 4B1–B3). After exposure to CA, the fluorescence in the cell walls was uneven and the fluorescence in the septa became weaker (Figure 4B4–B6).

### Effect of CA on the Chitin Contents of *G. citri-aurantii*

The chitin contents of *G. citri-aurantii* mycelia rapidly decreased (*P* < 0.05) with the CA treatment before 60 min of exposure. After 120 min of exposure, the chitin content in the CA treated samples was 8.25 ± 0.36%, which was significantly lower than the control samples (12.06 ± 0.80%) (*P* < 0.05) (Figure 4C).

### Effects of CA on the Gene Expressions Involved in the Cell Wall Integrity

**FIGURE 5:**
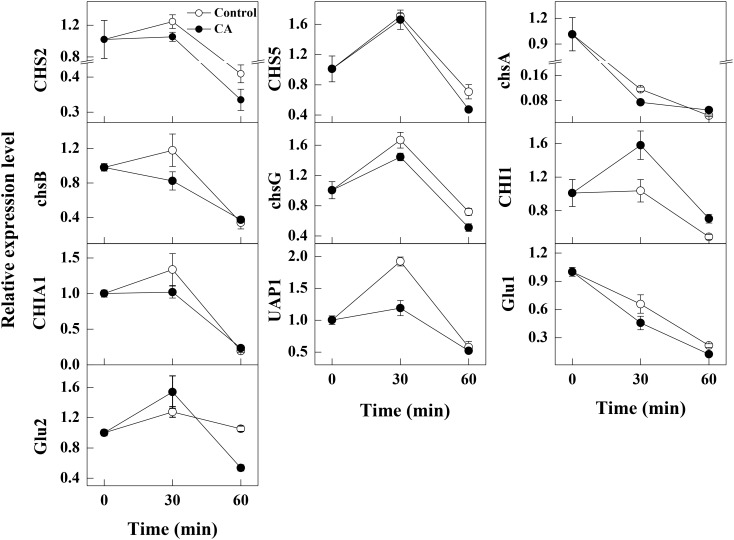
Changes in the expression levels of the cell wall integrity related genes in control and 1/2MIC CA treated *G. citri-aurantii* mycelia. The data presented are the means of pooled data. Error bars indicate the SDs of the means (*n* = 3).

After 30 min of exposure to CA, the expression levels of *CHS2, chsA, chsB, chsG*, and *UAP1* involved in chitin biosynthesis were significantly lower than the control samples. By contrast, the expression level of *CHI1* related to chitin decomposition in CA treatments was significantly higher than the control sample at 30 min of incubation. Moreover, the expression levels of *chsA, chsB* and *UAP1* in CA treated samples were equal to those in control samples at 60 min of incubation, whereas the gene expression of *CHS2, CHS5* and *chsG* were all repressed by CA treatment at this time. The expression level of another gene *CHIA1* involved in chitin decomposition had no difference between CA treatments and the control samples. The gene expression levels of *endo-1,3(4)-β-glucanase* (*Glu1*) and *glucan 1,3-β-glucosidase* (*Glu2*), associated with glucans hydrolysis, were significantly lower than the control groups at 30 and 60 min of exposure, respectively (Figure 5).

## Discussion

The lack of registered fungicides in controlling *G. citri-aurantii* infections in citrus fruit promotes the need to explore new sources of antifungal substances. Essential oils are promising alternatives to conventional fungicides for controlling postharvest diseases (Talibi et al., 2012; Shao et al., 2013, 2015; Regnier et al., 2014; Wei et al., 2018). Previously, CA was demonstrated to be effective in controlling some postharvest pathogens, including *P. digitatum* and *G. citri-aurantii*, and decrease the disease index of sour rot and green mold decay of citrus (Wu et al., 2017; Duan et al., 2018). This study aims to explore the possible antifungal mechanism of CA against the mycelial growth of *G. citri-aurantii*. In our study, CA caused distorted, shriveled, and squashed hyphae, and structural disorganization of the cytoplasm, which was consistent with the previous literature that essential oils could elicit versatile roles on cell walls, cell membranes, and cytoplasmic contents (Xie et al., 2004; Tao et al., 2014; Xing et al., 2014; Zhou et al., 2014; Shao et al., 2015; Wei et al., 2018).

The integrity of the plasma membrane played a crucial role in maintaining fungal viability (Shao et al., 2013; Tao et al., 2014). Earlier studies demonstrated that essential oils could increase the cell membrane permeability and damage the membrane integrity by decreasing the important cell membrane structure components, total lipids and ergosterol (Shreaz et al., 2011; Tao et al., 2014; OuYang et al., 2016). Shreaz et al. (2011) reported that CA exerted its antifungal activity by targeting the sterol biosynthesis of *Candida*. However, in our study, the addition of CA did not decrease the contents of total lipids and ergosterol of *G. citri-aurantii*. Interestingly, CA induced the massive accumulation of total lipids before 60 min of exposure (Figure 3A). This might lead to the thickening of cell membranes and reduce the amount of PI that enters cell membrane and thus decreased the fluorescence intensity. After 120 min of exposure, the total lipid content in CA treated group was rapidly decreased but remained at a similar level to that of control group. Correspondingly, the PI stain experiment results showed that the membrane integrity was significantly disrupted. These results suggested that the antifungal activity of CA is likely not caused by damage to the membrane permeability and integrity. This discrepancy may be caused by a difference in the structure of different fungal species. For example, citral was reported to inhibit the ergosterol biosynthesis of *P. italicum* and *P. digitatum* (Tao et al., 2014; OuYang et al., 2016), whereas the ergosterol content of *Candida albicans* was not influenced by citral (Leite et al., 2014).

The fungal wall is a flexible structure that provides protection to the cell and therefore is crucial for fungal permeability (Klis, 1994). The cell wall structure of fungi may be severely affected by essential oils including *Hyssopus officinalis* oil and tea tree oil (Ghfir et al., 1997; Shao et al., 2013). AKP is an enzyme produced in the cytoplasm and leaked into the periplasmic space. Generally, AKP releases from fungal cells with impaired cell wall permeability (Yang et al., 2016). In a previous study, tea tree oil was reported to cause the leakage of AKP and thus damaged the cell wall integrity of *Botrytis cinerea* (Shao et al., 2013). In this study, a significantly higher AKP activity was also observed in CA treatments, indicating that CA disrupted the cell wall permeability of *G. citri-aurantii*. Chitin, a *β*-(1,4)-linked polymer of N-acetylglucosamine, is an important structural component in the cell walls of filamentous fungal that plays a critical role in fungi development and pathogenicity (Klis, 1994). The fluorochrome stain calcofluor binds preferentially to areas containing chitin and is widely used to determine cell wall integrity (Lewtak et al., 2014). In our study, a weaker and uneven fluorescence in the cell walls, particularly in the septa, was observed, which indicated that the chitin content in the cell walls may be affected by CA treatment. This result was also confirmed by the chitin contents assay. These results demonstrated that the cell wall was an important antifungal target of CA.

In fungi, the chitin content is genetically regulated by some chitin synthase genes. Chitin synthases (CHS2, CHS5, chsA, chsB, and chsG) are located on the outer surface of the fungal mycelia and are responsible for the hyphal growth and conidiation (Mellado et al., 1996; Ichinomiya et al., 2002). Among them, *CHS2* and *chsA* are closely related with cell wall integrity (Fujiwara et al., 2000; Soulie et al., 2003). In addition, *CHS2* is responsible for the synthesis of septal chitin and serves indispensable roles in the formation of the primary septum (Fujiwara et al., 2000; Schmidt et al., 2002). *CHS5* is required for maintaining the normal levels of chitin in *S. cerevisiae*, and mutants lacking *CHS5* have a 75% reduction in cell wall chitin and altered morphology (Santos et al., 1997). As revealed in this study, the expression levels of the tested chitin synthases genes related to chitin synthesis were all repressed by CA treatment, suggesting that the build of cell wall might be destructed. This has been reported in previous studies that CA served as a cell wall target antifungal agent against *S. cerevisiae* cells by the inhibition of cell wall-synthesizing enzymes, such as *β*-(1,3)-glucan synthase and chitin synthase I (Bang et al., 2000; Shreaz et al., 2016). Meanwhile, the gene *UAP1* responsible for producing the essential initial substrate (UDP-N-acetylglucosamine) in chitin biosynthesis, and its expression level was decreased after CA treatment, which indicated that the biosynthesis of UDP-N-acetylglucosamine and chitin might be impaired, as reported by another study (Maiti et al., 2015). Furthermore, the gene expression of *CHI1*, a gene related to the hydrolysis of chitin, was stimulated, and this may accelerate the hydrolysis of chitin and therefore led to the breakdown of cell wall integrity (Takaya et al., 1998; Zhang et al., 2017). In our study, the increase of extracellular AKP activity and the decrease of chitin content also supported the hypothesis that CA inhibited the growth of *G. citri-aurantii* by affecting the formation of cell wall and the integrity of cell wall.

Meanwhile, glucans are also the components constituting the cell wall. Therefore, the structure and stability of fungal cell is also influenced by such cell wall degrading enzymes as Glu1 and Glu2 involved in glucan metabolism. These two enzymes are both *β*-1,3-glucan catabolic enzymes responsible for the hydrolysis of beta-D-glucose (Matsui et al., 2017). In our study, the expression levels of *Glu1* and *Glu2* were significantly lower than the control samples, indicating the metabolism of glucans might be greatly affected by the CA treatment. These results indicated that CA treatment not only inhibited chitin synthesis but also induced the hydrolysis of chitins, thereby affecting the formation of cell wall and destroying the cell integrity of *G. citri-aurantii*.

## Conclusion

In conclusion, our results showed that the impairment of cell wall caused by CA was prior to the damage of cell membrane, indicating that the main antifungal mechanism of CA against *G. citri-aurantii* is attributed to the impairment to the formation of cell wall and the disruption in cell wall integrity.

## Author Contributions

NT designed the research. QO performed the research. NT and QO analyzed the data. QO, XD, LL, and NT wrote the manuscript. All authors contributed to study design and provided input on the manuscript preparation, and given approval to the final version of the manuscript.

## Conflict of Interest Statement

The authors declare that the research was conducted in the absence of any commercial or financial relationships that could be construed as a potential conflict of interest. The reviewer JH and handling Editor declared their shared affiliation.
